# Outcomes of the Surgical Stone Management in Pelvic Ectopic Kidneys: A Retrospective Comparison of Three Different Approaches

**DOI:** 10.3390/jcm14062081

**Published:** 2025-03-19

**Authors:** Fatih Bicaklioglu, Mahmut Selman Mert, Resul Sobay, Ozgur Arikan, Mehmet Erhan Aydin, Mehmet Uslu, Salih Yildirim, Kemal Sarica

**Affiliations:** 1 Department of Urology, Kartal Dr. Lutfi Kirdar City Hospital, Istanbul 34865, Türkiye; mselmanm-00@hotmail.com; 2 Department of Urology, Istanbul Umraniye Training and Research Hospital, University of Health Sciences, Istanbul 34766, Türkiye; drresulsobay@gmail.com; 3 Department of Urology, Goztepe Prof. Dr. Suleyman Yalcın City Hospital, Medeniyet University, Istanbul 34722, Türkiye; arikanozgur@hotmail.com; 4 Department of Urology, Eskisehir City Hospital, Eskisehir 26080, Türkiye; merhanaydin@gmail.com; 5 Department of Urology, Kafkas University School of Medicine, Kars 36100, Türkiye; drmhmtuslu@gmail.com; 6 Department of Urology, Sancaktepe Sehit Prof. Dr. Ilhan Varank Training and Research Hospital, Istanbul 34098, Türkiye; yldrmsalih7@gmail.com (S.Y.); saricakemal@gmail.com (K.S.)

**Keywords:** pelvic ectopic kidney, kidney stone, renal ectopia, open pyelolithotomy, laparoscopic pyelolithotomy, retrograde intrarenal surgery

## Abstract

**Background/Objectives**: This study evaluates and compares the surgical outcomes of open pyelolithotomy, laparoscopic pyelolithotomy, and retrograde intrarenal surgery (RIRS) in the management of pelvic ectopic kidney stones. **Methods**: A retrospective analysis was conducted on 47 adult patients with pelvic ectopic kidney stones who underwent surgery between January 2009 and January 2024. Patients were categorized as open pyelolithotomy (n = 15), laparoscopic pyelolithotomy (n = 14), or RIRS (n = 18). Stone-free (SF) rates were assessed in the early postoperative period (1st or 2nd day), in the 1st month, and in the 3rd month. Demographic data, stone characteristics, operative data, and complications were recorded. **Results**: RIRS had significantly shorter operative and hospitalization times but a lower SF rate in the 3rd month (44.4%) compared to laparoscopy (92.9%) and open pyelolithotomy (86.7%). Additional procedures were required in 50% of RIRS cases to achieve SF status, while none were needed in the other groups. Complications included three Grade 2 cases (two bleeding; transient creatinine elevation) in open pyelolithotomy, two Grade 2 (urinary leakage; infection) and two Grade 3 cases (conversion to open surgery; trapped stent removal) in laparoscopic pyelolithotomy, and one Grade 2 case (febrile infection) in RIRS. **Conclusions**: Laparoscopic pyelolithotomy demonstrated the highest efficacy and comparable complication rates; making it the preferred approach for pelvic ectopic kidney stones. Open pyelolithotomy remains a valuable alternative where laparoscopic expertise or resources are limited. Although less invasive, RIRS showed lower efficacy due to the challenging anatomy of pelvic ectopic kidneys.

## 1. Introduction

Congenital anomalies of the kidney and urinary tract (CAKUT) include a variety of developmental abnormalities resulting from malformations of the kidneys and their outflow tracts. These anomalies represent a significant public health burden, affecting between 4 and 60 of each 10,000 live births and representing 20% of adult-onset end-stage renal disease (ESRD) [1]. In addition, CAKUT is an important cause of renal failure, occurring in 40–50% of pediatric patients and 7% of adults suffering from chronic renal failure. CAKUT disorders include nephropathies such as renal aplasia, hypoplasia, dysplasia, oligomeganephronia, ectopic kidneys, and horseshoe kidneys, among which pelvic ectopic kidney is a particularly rare congenital anomaly [2]. Pelvic ectopic kidneys, which are located anterior to the sacrum and below the aortic bifurcation, have a reported incidence of 1 in 2200 to 1 in 3000 in autopsy series [3,4]. The management of stones in these cases is more challenging compared to kidneys in their normal anatomical position due to factors such as abnormal anatomy, aberrant vascular structures, the presence of surrounding intestinal structures, and the kinked ureter with its high insertion into the renal pelvis.

Before the advent of endourology, open surgery was the only available treatment option. However, significant advancements in instrument technology have led to the widespread adoption of minimally invasive and noninvasive techniques. In this context, shockwave lithotripsy (SWL), the sole noninvasive option, has been safely used for managing these stones, though achieving a completely stone-free status remains highly limited [5,6]. Subsequently, advancements in endourology led to the development of more effective techniques, including laparoscopy-assisted PNL by Eshghi et al. [7], laparoscopic pyelolithotomy by Harmon et al. [8], and PNL with ultrasonographic access by Desai et al. [9]. Furthermore, advancements in ureteroscopic stone treatment have enabled the application of retrograde intrarenal surgery (RIRS) using fine scopes as a less invasive alternative for pelvic ectopic kidney stones [10]. More recently, robotic-assisted laparoscopic pyelolithotomy has emerged as a feasible option for managing these cases [11]. Despite these advancements, there remains a lack of consensus on the optimal surgical approach for managing stones in pelvic ectopic kidneys. EAU guidelines on urolithiasis provide no specific recommendations for the management of stones in pelvic ectopic kidneys; instead, SWL, RIRS, PNL, and laparoscopic surgery are presented as potential treatment options [12].

Since no surgical approach has been established as superior, comparative analyses of different techniques are crucial for guiding clinical decision-making. Over the past 15 years, we have primarily utilized open pyelolithotomy, laparoscopic pyelolithotomy, and RIRS for the surgical treatment of pelvic ectopic kidney stones. As this study is retrospective, we aimed to evaluate and compare the outcomes of these three techniques. By analyzing perioperative parameters and clinical outcomes, this study seeks to provide valuable insights into the efficacy and safety of these approaches in this rare and anatomically complex condition.

## 2. Materials and Methods

### 2.1. Patients

This retrospective study used the medical records of 47 adult patients (>18 years) with pelvic ectopic kidney stones who underwent surgical treatment between January 2009 and January 2024.

Inclusion criteria: Adult patients (≥18 years) with pelvic ectopic kidney stones who underwent surgical intervention between January 2009 and January 2024.

Exclusion criteria: Patients younger than 18 years.

### 2.2. Ethical Aspects

The study protocol was approved by the Institutional Ethics Committee of Kartal Dr. Lutfi Kirdar City Hospital (protocol code 2024/010.99/3/21 and date of approval: 29 April 2024).

### 2.3. Study Design

This was a retrospective, comparative study evaluating the surgical outcomes of different treatment modalities for pelvic ectopic kidney stones over 15 years. Patients were categorized into three groups based on the surgical approach:

− Open pyelolithotomy (n: 15);
− Laparoscopic pyelolithotomy (n: 14);
− Retrograde intrarenal surgery (n: 18).

The choice of surgical approach was based on real-world clinical decision-making rather than a standardized protocol. Multiple factors, including stone size, location, patient-specific anatomical characteristics, and prior surgical history, influenced the selection of treatment modality. In particular, patients with smaller stones (<15 mm) were more likely to undergo RIRS, whereas larger or more complex stones were managed with open or laparoscopic pyelolithotomy. Additionally, the surgeon’s experience played a key role in the decision-making process, as those with limited laparoscopic expertise were more likely to perform open pyelolithotomy. In our clinical experience, none of the patients underwent laparoscopy-assisted or pure percutaneous nephrolithotomy (PNL) or robotic-assisted laparoscopic pyelolithotomy. Therefore, these techniques were not included in the study.

### 2.4. Study Outcomes

The primary outcome of the study was the stone-free (SF) rate, assessed in the 3rd month using non-contrast-enhanced computed tomography (NCCT), which served as the most sensitive method for detecting residual fragments.

The secondary outcomes of the study included several perioperative and postoperative parameters. Complication rates were evaluated and classified according to the Clavien–Dindo classification to assess the safety of each surgical approach [13]. Hospitalization time (measured in days) was recorded to compare the postoperative recovery periods among the different surgical techniques. Operation time (measured in minutes) was analyzed to evaluate the efficiency of each procedure. Additionally, the need for additional interventions, including secondary procedures for residual stones, was documented to determine the effectiveness of each surgical approach in achieving complete stone clearance.

These outcomes were evaluated comparatively across the three surgical groups, providing insights into the effectiveness and safety profile of each approach.

### 2.5. Preoperative Evaluation

**Figure 1:**
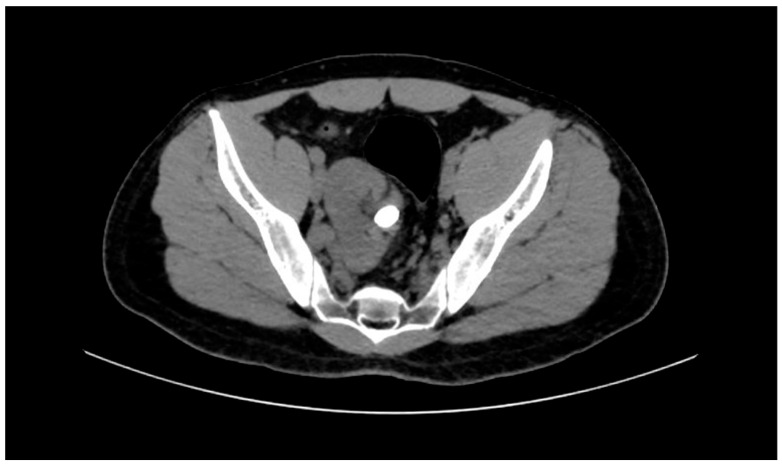
Preoperative NCCT image of a patient with a pelvic ectopic kidney stone. The scan reveals the stone location and anatomical positioning of the pelvic ectopic kidney, which are essential factors in determining the appropriate surgical approach.

All patients underwent a standard preoperative evaluation, including laboratory tests such as complete blood count, serum creatinine, liver function tests, coagulation profile, urinalysis, and urine culture. Non-contrast-enhanced computed tomography (NCCT) was performed on all patients as part of the preoperative imaging assessment (Figure 1). NCCT was utilized to evaluate stone size, stone location within the kidney (e.g., renal pelvis, lower calyx, multiple calyces), stone complexity (e.g., staghorn calculus, solitary pelvic stone), and Hounsfield unit (HU) to estimate stone density and composition. Additionally, NCCT provided detailed anatomical information regarding the pelvic ectopic kidney’s position, orientation, and collecting system anatomy, which were critical for surgical planning. Patients with urinary tract infections in the preoperative period underwent surgery only after receiving appropriate antibiotic treatment.

### 2.6. Surgical Procedure

***Open pyelolithotomy:*** Patients were positioned in the supine position. A midline subumbilical incision was made, and the abdomen was entered via a transperitoneal approach. The intestines were carefully retracted to expose the pelvic ectopic kidney. The posterior peritoneum was then opened to access the kidney, and the renal pelvis was carefully dissected. A pyelolithotomy was performed to extract the stone, after which the pyelolithotomy incision was closed using Polyglactin 910 (Vycril^®^ 4/0, Ethicon, Johnson & Johnson, Diegem, Belgium) sutures. A Double-J (D/J) stent (PlastiMed^®^, Ostim, Türkiye) was placed at the discretion of the surgeon. The peritoneal layer was reapproximated, a drain was placed in the surgical field, and the incision was closed in anatomical layers.

**Figure 2:**
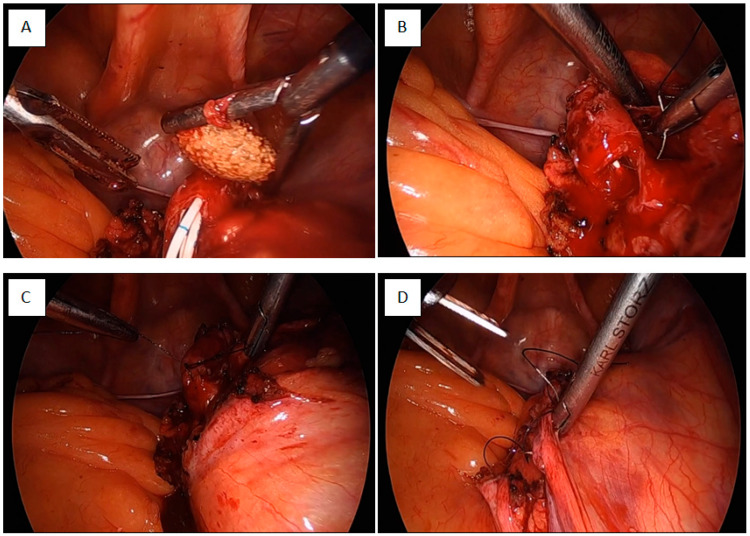
Magnification of laparoscopic pyelolithotomy. (**A**) Removal of renal pelvis stone from pelvic ectopic kidney; (**B**,**C**) laparoscopic suturing of pyelolithotomy line; (**D**) laparoscopic suturing of parietal peritoneum on pelvic ectopic kidney.

***Laparoscopic pyelolithotomy:*** Initially, patients were placed in the lithotomy position for retrograde D/J stent (PlastiMed^®^, Ostim, Türkiye) placement. After stent insertion, they were repositioned to the supine Trendelenburg position to facilitate bowel displacement away from the surgical field. A three-port laparoscopic technique was used. The first trocar was inserted a few centimeters above the umbilicus using the Veress needle technique for pneumoperitoneum creation, while the remaining two trocars were placed under direct vision. Following adequate bowel retraction, the posterior peritoneum was opened, and the renal pelvis was carefully dissected. A pyelolithotomy was performed, and the stone was extracted and pyelolithotomy incision was closed using Polyglactin 910 (Vycril^®^ 4/0, Ethicon, Johnson & Johnson, Diegem, Belgium) sutures. An endobag was placed for stone retrieval. After placing the drain, the stone was removed through the camera port, which had been widened to accommodate the extraction. The trocar sites were then closed appropriately. Figure 2 illustrates the various stages of the procedure.

Open and laparoscopic pyelolithotomy procedures did not include intrarenal endoscopy via the pyelolithotomy incision.

***Retrograde intrarenal surgery:*** Patients were positioned in the lithotomy position for the procedure. A guidewire was placed using a semirigid ureterorenoscope. Subsequently, a ureteral access sheath was introduced over the guidewire to facilitate flexible ureteroscopic manipulation. In cases where a narrow ureter or ureteral orifice stricture prevented ureteroscopic advancement, a D/J stent (PlastiMed^®^, Ostim, Türkiye) was placed, and the procedure was postponed for one month to allow passive ureteral dilation. A flexible ureteroscope was advanced through the access sheath to reach the stone, and holmium: YAG laser lithotripsy was performed using a 272 µm laser fiber in a dusting technique at 0.6 Joules and 10 Hz to fragment the stone into fine particles, promoting spontaneous passage. At the end of the procedure, the flexible ureteroscope was withdrawn under direct visualization, allowing for careful inspection of the ureter to identify any potential injuries. Finally, a D/J stent was placed using a semirigid ureteroscope.

### 2.7. Clinical and Perioperative Data Collection

Demographic data such as age, gender, history of previous surgery, additional comorbidities, and serum biochemistry results were recorded. Stone-related parameters, including side, size, and Hounsfield unit (HU) were documented, as well as surgical information such as operation time, complications (the Clavien–Dindo classification), hospitalization time, stone-free (SF) rates at various time points (early postoperative period, 1st month, and 3rd month), and the need for additional interventions.

### 2.8. Follow-Up and Outcome Assessment

Postoperative imaging for residual stones was performed using kidney, ureter, and bladder (KUB) radiography on the 1st or 2nd postoperative day for the early postoperative period, KUB combined with ultrasonography (USG) at the 1st month, and non-contrast-enhanced computed tomography (NCCT) at the 3rd month. While KUB and USG provide an initial assessment of stone clearance, NCCT at 3 months serves as the most sensitive and definitive method for detecting residual fragments. In this study, stone-free status was defined as the complete absence of residual stone fragments. Clinically insignificant residual fragments (CIRF) were defined as asymptomatic, non-obstructive stone fragments ≤ 4 mm that did not cause infection. Failure was defined as residual stone fragments > 4 mm or symptomatic fragments (≤4 mm) causing obstruction or infection.

### 2.9. Statistical Analysis

Statistical analysis was performed using IBM SPSS Statistics version 21 (IBM Corp., Armonk, NY, USA). The Shapiro–Wilk test was used to assess the normality of data distribution. Continuous variables were analyzed using the ANOVA test for normally distributed data, while the Kruskal–Wallis test was applied for non-normally distributed data. Categorical variables were compared using the chi-square test, and Fisher’s exact test was employed when appropriate. A *p*-value of <0.05 was considered statistically significant.

A post hoc power analysis was conducted using GPower (ANOVA, fixed effects, omnibus, one-way) to assess the statistical power for comparing stone-free rates among the three surgical groups (open pyelolithotomy, laparoscopic pyelolithotomy, and RIRS) at the 3rd postoperative month. The analysis demonstrated a power of 0.85 with a significance level of 0.05 and an effect size of 0.50, confirming that the study had sufficient statistical power for this comparison.

## 3. Results

A total of 47 patients (37 males and 10 females) with pelvic ectopic kidneys underwent one of three different surgical interventions for stone removal. The age of the patients ranged from 18 to 74 years, with a mean of 42.19 ± 17.11 years. The baseline characteristics of the patients are summarized in Table 1. There were no statistically significant differences in terms of age or comorbidities between the three groups.

Stone-related characteristics and surgical intervention details are provided in Table 2. RIRS was more commonly performed in patients with relatively smaller stones (*p* < 0.001). Additionally, the RIRS group demonstrated shorter operative times (*p* < 0.001) and hospitalization times (*p* < 0.001) compared to the other groups. However, the need for additional procedures to achieve stone-free status was significantly higher in the RIRS group (*p* < 0.001).

The stone-free (SF) rates at the 3rd postoperative month, including cases requiring a second RIRS session, were 86.7% for open pyelolithotomy, 92.9% for laparoscopic pyelolithotomy, and 44.4% for RIRS, with the SF rate being significantly lower in the RIRS group compared to the other groups (*p* = 0.005). When assessing the SF rates at the 1st postoperative month for open pyelolithotomy, laparoscopic pyelolithotomy, and single-session RIRS, the rates were 80.0%, 92.9%, and 27.9%, respectively, with the RIRS group demonstrating significantly lower SF rates (*p* < 0.001). In the open and laparoscopic pyelolithotomy groups, failure was primarily observed in patients with stones located in both the renal pelvis and a single calyx. While the pelvic stones were successfully removed in these cases, residual calyceal stones persisted. Notably, in the open pyelolithotomy group, one patient with a 4.5 mm residual stone spontaneously expelled the fragment postoperatively and was subsequently reclassified as stone-free at the 3-month follow-up. No statistically significant differences were observed between the SF rates of open and laparoscopic pyelolithotomy groups at the 3rd postoperative month (*p* = 1). Detailed SF rate comparisons are shown in Table 3.

An evaluation of complications revealed several notable findings across the three surgical groups. In the open pyelolithotomy group, two patients experienced intraoperative bleeding that required blood transfusion, and one patient developed transient creatinine elevation, which resolved with intravenous fluid therapy; all these cases were classified as Clavien–Dindo Grade 2. In the laparoscopic pyelolithotomy group, one patient was hospitalized for urinary leakage and fever, which were managed conservatively (Clavien–Dindo Grade 2), while another patient required hospitalization and intravenous antibiotic therapy due to postoperative fever and a resistant urinary infection (Clavien–Dindo Grade 2). Additionally, one patient in this group required conversion to open surgery due to intraoperative complications (Clavien–Dindo Grade 3). Another patient underwent diagnostic ureteroscopy for a double-J stent that became trapped in the pyelolithotomy suture line; the suture was successfully cut using a Holmium-YAG laser, and the stent was removed without further complications (Clavien–Dindo Grade 3). In the RIRS group, one patient developed a postoperative fever that necessitated hospitalization and intravenous antibiotic therapy, classified as Clavien–Dindo Grade 2.

## 4. Discussion

Stone formation in pelvic ectopic kidneys is an uncommon condition due to the rarity of this congenital anomaly. The treatment of stones in such cases remains controversial, as limited data are available in the literature. Current treatment options include open surgery, shockwave lithotripsy (SWL), laparoscopy-assisted percutaneous nephrolithotomy (PNL), PNL, laparoscopic surgery (conventional or robot-assisted), and retrograde intrarenal surgery (RIRS). Despite these options, the outcomes of stone removal procedures in pelvic ectopic kidneys are not well-documented.

Baltacı et al. reported a 25% stone-free (SF) rate following SWL in 4 cases, while Kupeli et al. achieved a 54% SF rate in 13 patients at the 3rd monthly follow-up [5,6]. Expanding on Kupeli’s findings, Tunc et al. conducted the largest series with 14 pelvic ectopic kidney cases, reporting an SF rate of 57.2% [14]. However, a more recent study by Ahmad et al. further highlights the limited efficacy of SWL in ectopic kidneys, reporting a 0% SF rate in 3 pelvic ectopic kidney cases, compared to 75% in lumbar ectopic kidneys and 100% in abdominal ectopic kidneys [15]. These findings underscore the unique anatomical challenges of pelvic ectopic kidneys. As a result, SWL has become a less favorable treatment option for pelvic ectopic kidney stones, and we have not utilized this technique in our clinical practice.

Laparoscopy-assisted PNL, first described by Eshghi et al., involved displacing intestinal loops surrounding the kidney to safely access stones while minimizing the risk of injury to adjacent organs [7]. Holman and Tóth later refined this technique in 15 cases of pelvic dystopic kidneys, using laparoscopic and fluoroscopic guidance to establish an antegrade nephrostomy track. Their method achieved high success with complete stone clearance and no major complications, making it a safe, minimally invasive option [16]. Later, Desai et al. demonstrated that pure supine PNL under ultrasonographic guidance could be employed in such cases. However, the atypical anatomy and associated risks of organ or vascular injury have limited the widespread use of this approach [9]. More recently, Otano et al. evaluated ultrasound-guided PNL in 26 patients with pelvic ectopic kidneys, achieving an 88% stone-free rate with minimal complications (four Clavien grade 1, one Clavien grade 2, and one Clavien grade 3). Their study demonstrated that ultrasound guidance could provide a safe and effective approach to percutaneous access in such anatomically challenging cases when performed by experienced hands [17]. In our clinical experience, neither laparoscopy-assisted nor pure PNL procedures were performed for the treatment of pelvic ectopic kidney stones.

With advancements in laparoscopic techniques, laparoscopic pyelolithotomy has emerged as a practical, purely laparoscopic option. However, this approach has limitations, particularly in cases with significant inflammation, aberrant vascular anatomy, or a small intrarenal pelvis, which make dissection and pyelolithotomy challenging, especially if the surgeon lacks experience in advanced laparoscopy. In such cases, laparoscopy-assisted PNL remains a viable alternative to ensure complete stone clearance.

In our series of 47 patients, 18 underwent RIRS, 14 laparoscopic pyelolithotomy, and 15 open pyelolithotomy. Literature reviews reveal no series specifically addressing open pyelolithotomy for ectopic pelvic kidney stones. However, as highlighted in a review by El Husseiny and the EAU guidelines, open surgery is a reasonable alternative for stones in ectopic kidneys where percutaneous access or SWL is challenging or infeasible [18]. Open surgery is also more commonly performed in developing countries, as shown by Patandung and Hadisurya, who reported frequent use of open pyelolithotomy for ectopic kidney stones in Indonesia [19,20]. In our study, open pyelolithotomy achieved an 86.7% SF rate in the 3rd month, with an average hospitalization duration of 4.8 ± 1.3 days and a 20% Clavien–Dindo Grade 2 complication rate. However, retrospective limitations prevented us from assessing postoperative pain or recovery, precluding a direct comparison of recovery outcomes with other groups.

Among the 14 patients who underwent laparoscopic pyelolithotomy, the SF rate was 92.9% in the 3rd month, with a 28.5% complication rate (two Clavien Grade 2, two Clavien Grade 3), and an average hospitalization period of 3.9 ± 2.6 days. Comparable findings were reported by Ramakumar et al., who achieved a 100% SF rate but had a 27% conversion to open surgery [21]. Elbahnasy et al. reported a 90.9% SF rate with an average hospitalization time of 4.5 days and complications (urinary leakage) in 2 cases [22]. Ergin et al. achieved a 100% SF rate in 9 cases with a shorter hospitalization time (1.9 ± 0.4 days) and no complications [23]. Similarly, a larger series of 15 patients by Aggarwal et al. reported a 93.3% SF rate, which increased to 100% after a single session of ESWL for one patient with a residual 8 mm caliceal stone. Their study showed a mean hospitalization time of 4.5 days (range: 4–7 days) and a mean operative time of 125 min (range: 90–190 min). Only one patient experienced a postoperative complication, a transient ileus lasting 48 h, managed conservatively [24]. These findings align with our results, though our complication rate was higher, likely due to variations in laparoscopic experience among surgeons. Notably, similar to Aggarwal et al.’s series, our study also included a case with residual caliceal stone, which we classified as a failure. As demonstrated in previous reports, such as the laparoscopic pyelolithotomy cases described by Fariña Pérez et al., the use of flexible nephroscopy through the pyelolithotomy incision can facilitate the removal of residual caliceal stones [25]. This approach provides a minimally invasive solution for effectively managing caliceal stones, potentially reducing the need for additional procedures and improving overall stone-free rates. Moreover, robotic-assisted surgery has emerged as an alternative to conventional laparoscopy, offering enhanced dexterity and precision. The improved 3D visualization and the articulating capabilities of robotic instruments may allow for more efficient stone extraction and precise pelvic reconstruction, potentially shortening operative time and improving outcomes [11,26]. However, despite these advantages, the high cost associated with robotic systems remains a significant limitation, restricting its widespread adoption.

For the 18 patients who underwent RIRS, the SF rate at the 3rd postoperative month was 44.4%, including second-session RIRS cases. The complication rate was 5.5% (one Clavien Grade 2) with an average hospitalization duration of 1.8 ± 1.9 days. In contrast, Weizer et al. reported a 75% SF rate in their first series of 4 RIRS cases [10], and Ergin et al. achieved an 83.6% SF rate in 33 cases [23]. The relatively lower SF rate in our RIRS group may be attributed to differences in the definition of stone-free status across studies. While our study defined SF as no residual stones, other studies considered residual fragments < 5 mm or <2 mm as SF, which could lead to variations in reported outcomes. For example, Bozkurt et al. reported an 84.7% success rate using a stone-free or <2 mm residual fragment definition, while Geavlete et al. reported SF rates of 60.1%, 84.1%, and 94.4% after the first, second, and third RIRS sessions, respectively, using a <3 mm residual fragment criterion [27,28]. Legemate et al. reported a lower SF rate (20%) in ectopic kidneys, which aligns with our findings, using stricter criteria (no fragments >1 mm on NCCT) [29]. Ozmerdiven et al. reported the outcomes of RIRS in 7 patients with pelvic ectopic kidneys, defining stone-free status similarly to our study as the absence of residual fragments. At the first postoperative month, a stone-free rate of 43% (3 out of 7 patients) was achieved. However, following a second RIRS session in one patient and pyelolithotomy in two patients, the stone-free rate increased to 71.5% (5 out of 7 patients) by the third postoperative month. When excluding the contribution of pyelolithotomies to stone-free status and focusing solely on RIRS outcomes, we observe that the stone-free rates are consistent with those reported in our study [30]. Most RIRS studies in pelvic ectopic kidneys have been conducted by experienced endourologists. In contrast, our study included surgeons with varying levels of experience. This difference may have contributed to the variation in stone-free rates across studies. To assess whether our results reflect the expected stone-free rates following RIRS, the Resorlu-Unsal Stone Score (RUSS) may be utilized as a practical tool, as it also includes patients with anomalous kidneys. This scoring system provides a simple and quick method for predicting stone-free rates after RIRS, aiding in both patient counseling and surgical decision-making for the management of renal stones. The RUSS score evaluates four parameters, assigning 1 point for each criterion, with a total score ranging from 0 to 4: stone size > 20 mm, lower pole location with an infundibulopelvic angle (IPA) < 45°, multiple stones in different calyces (>1), and abnormal renal anatomy [31]. The Resorlu-Unsal Stone Score (RUSS) has been externally validated in multiple studies, confirming its reliability in predicting stone-free rates after RIRS. Sfoungaristos et al. and Tufano et al. demonstrated that RUSS is a significant predictor of postoperative stone-free status [32,33]. In our series, the Resorlu-Unsal Stone Score (RUSS) was assessed in 18 patients. The majority had a score of 1 (9 patients, 50.0%), followed by a score of 2 (7 patients, 38.9%), while scores of 3 and 4 were each observed in 5.5% (1 patient) of cases. The mean RUSS score was 1.67 ± 0.84. In the study by Sfoungaristos et al., stone-free rates progressively decreased with an increasing Resorlu-Unsal Stone Score (RUSS). The stone-free rate was 85.4% for patients with a RUSS of 0, 70.8% for a score of 1, 50.0% for a score of 2, and 25.0% for scores of 3 or higher, demonstrating a negative correlation between higher RUSS and stone-free outcomes [32]. Considering this distribution, our 44.4% stone-free rate after RIRS in pelvic ectopic kidneys appears reasonable, as it aligns with the expected trend of decreasing stone-free rates with increasing RUSS scores. While scoring systems like RUSS help predict surgical outcomes, advancements in endourology continue to refine treatment success rates. Recent advancements in endourology, such as the flexible and navigable suction ureteral access sheath (FANS), have shown promising results in improving SF rates in RIRS. In a randomized controlled trial, FANS demonstrated a significantly higher SF rate (95% vs. 67%) compared to conventional access sheaths, along with reduced re-intervention rates and shorter operative times [34]. Similarly, the introduction of thulium fiber laser (TFL) technology has provided advantages over the traditional holmium laser, offering higher ablation efficiency, reduced retropulsion, and improved fragmentation speeds, leading to potentially better outcomes in RIRS [35]. However, while these innovations are becoming more widely adopted in endourology, studies specifically evaluating their efficacy in rare anatomical conditions, such as pelvic ectopic kidneys, remain lacking. Prospective studies focusing on RIRS outcomes in pelvic ectopic kidneys with FANS and TFL could provide valuable insights.

Overall, our findings are that laparoscopic and open pyelolithotomy achieve comparable SF rates, whereas RIRS results in significantly lower SF rates. The primary reason for this differences is that pyelolithotomy allows for complete stone removal without fragmentation, making it easier to achieve an SF status. In contrast, the limitations of flexible ureteroscopy in accessing all stone locations contributed to the lower SF rates observed in RIRS cases. While laparoscopic pyelolithotomy provided a comparable SF rate to open surgery, it also resulted in shorter hospital stays and faster recovery. However, open pyelolithotomy remains a highly effective option, particularly for complex cases, though it is associated with increased postoperative pain and a longer return to daily activities. The magnification offered by laparoscopy also facilitates safer dissection of aberrant vascular structures and surrounding tissues. Despite these advantages, laparoscopic surgery required longer operative times and had a higher complication rate compared to RIRS. RIRS, while having lower efficacy, remains the least invasive option and may be preferable in patients with smaller stones or those unfit for more invasive surgery. Ultimately, the choice of technique should be guided by patient characteristics, stone burden, and available surgical expertise, balancing the benefits of minimally invasive approaches against the effectiveness of complete stone clearance.

This study has several limitations. First, its retrospective design inherently limits the ability to control for potential confounders and biases. Second, the relatively small sample size may limit the generalizability of our findings. Third, the study does not include laparoscopic-assisted or pure percutaneous nephrolithotomy (PNL) techniques, which could have influenced the outcomes and provided additional comparative insights. Fourth, the lack of postoperative outcomes such as pain, recovery time, and analgesic use, could introduce variability into the results. Additionally, the absence of long-term follow-up data prevents an evaluation of recurrence rates or late complications. Finally, the involvement of multiple surgeons with varying levels of experience in different surgical techniques may have influenced operative times, complication rates, and surgical outcomes. Differences in expertise could have played a role in procedural success rates, particularly in complex cases requiring advanced technical skills.

## 5. Conclusions

Our findings, in conjunction with the existing literature, suggest that laparoscopic and open pyelolithotomy achieve higher stone-free rates compared to RIRS in the management of pelvic ectopic kidney stones. Between these two approaches, laparoscopic pyelolithotomy is preferable due to its association with less postoperative pain, faster recovery, comparable complication rates, and similar stone-free outcomes. Nevertheless, open pyelolithotomy remains a valuable alternative, particularly in cases where laparoscopic expertise or equipment is unavailable, as it offers similar stone-free and complication rates.

## Figures and Tables

**Table 1:** Patient characteristics.

		RIRS (n: 18)	Laparoscopic Pyelolithotomy (n: 14)	Open Pyelolithotomy (n: 15)	*p*-Value
Age (mean)		44.7 ± 16.3	35.1 ± 14.7	45.6 ± 19	0.184 *
Gender	Male	12 (66.7%)	13 (92.9%)	12 (80%)	0.193 **
	Female	6 (33.3%)	1 (7.1%)	3 (20%)	
Comorbidity (diabetes, hypertension, etc.)		5 (27.7%)	6 (42.8%)	6 (40%)	0.633 ***
BMI (median [IQR])		24.8 (6.1)	22.2 (4.6)	25.5 (5.7)	0.027 ****
ASA classification					
ASA 1		10 (55.6%)	8 (57.1%)	9 (60.0%)	1.000 **
ASA 2		7 (38.9%)	5 (35.7%)	5 (33.3%)	
ASA 3		1 (5.6%)	1 (7.2%)	1 (6.7%)	
Previous SWL		4 (22.2%)	1 (7.1%)	1 (6.6%)	0.346 **
Previous stone surgery		5 (27.7%)	6 (42.8%)	9 (60%)	0.176 ***
Open pyelolithotomy		3	1	6	
RIRS		2	5	3	

* One-way ANOVA; ** Fisher’s exact test; *** chi-square; **** Kruskal–Wallis. Abbreviations: RIRS: retrograde intrarenal surgery; BMI: body mass index; ASA: American Society of Anesthesiologists; SWL: shockwave lithotripsy; IQR: interquartile range.

**Table 2:** Stone characteristics and surgical intervention-related data.

			RIRS (n: 18)	Laparoscopic Pyelolithotomy (n: 14)	Open Pyelolithotomy (n: 15)	*p*-Value
Stone size (mm) (median [IQR])			13.5 (8.0)	20.5 (7.0)	25 (8.0)	<0.001 *
Stone HU (mean)			920 ± 252	1083 ± 144	950 ± 251	0.120 **
Stone side	Right		7 (38.8%)	10 (71.4%)	13 (86.6%)	0.014 ***
	Left		11 (61.2%)	4 (28.6%)	2 (13.4%)	
Stone	Pelvis		10	9	8	
location	Lower calyx		3	2	1	
	Middle calyx		1	2	2	
	Upper calyx		1	-	-	
	Pelvis + one calyx		2	1	3	
	Partial staghorn		1	-	-	
	Staghorn		-	-	1	
Hospitalization time (day) (median [IQR])			1 (1.0)	3 (1.0)	4 (2.0)	<0.001 *
Operation time (min) (median [IQR])			65 (36.0)	100 (46.0)	95 (20.0)	<0.001 *
Post-operative complication			1 (5.5%)	4 (28.5%)	3 (20%)	0.199 ****
Requirement of additional intervention for stone-free		No	7 (38.8%)	13 (92.9%)	13 (86.7%)	<0.001 ****
		Yes	9 (50%)	0	0	
		Follow-up for asymptomatic calyx stone	2 (11.2%)	1 (7.1%)	2 (13.3%)	

* Kruskal–Wallis; ** one-way ANOVA; *** chi-square; **** Fisher’s exact test; Abbreviations: RIRS: retrograde intrarenal surgery; HU: Hounsfield unit: IQR: interquartile range.

**Table 3:** Stone-free status after surgery.

		Postoperative 1st or 2nd Day (KUB)	1st Month (KUB + USG)	3rd Month (NCCT)		
Open pyelolithotomy (n: 15)	-Stone-free -CIRF -Failure	12 (80%) - 3 (20%)	13 (86.7%) - 2 (13.3%)	13 (86.7%) - 2 (13.3%)	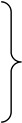	*p*-value = 1 *
Laparoscopic pyelolithotomy (n: 14)	-Stone-free -CIRF -Failure	13 (92.9%) - 1 (7.1%)	13 (92.9%) - 1 (7.1%)	13 (92.9%) - 1 (7.1%)		(3rd month open vs. laparoscopic pyelolithotomy)
RIRS (n: 18)	-Stone-free -CIRF -Failure	5 (27.9%) 1 (5.5%) 12 (66.6%)	7 (38.8%) - 11 (61.2%)	8 (44.4%) - 10 (55.6%)		
				[including 2nd RIRS] **		
***p*-value**		<0.001 *	0.001 *	0.005 *		

* Fisher’s exact test. ** After the first month of radiological evaluation of 18 RIRS patients, 7 patients were stone-free, 2 patients were followed up for asymptomatic calyx stones, 9 patients required additional intervention (7 patients had second session RIRS, 2 refused second session RIRS and lost follow-up). Abbreviations: KUB: kidney, ureter, and bladder; USG: ultrasonography; NCCT: non-contrast-enhanced computed tomography; RIRS: retrograde intrarenal surgery.

## Data Availability

The data supporting the findings of this study are available from the corresponding author upon reasonable request. Due to privacy and ethical restrictions, the dataset is not publicly accessible.

## Abbreviations

The following abbreviations are used in this manuscript:

CAKUT	congenital anomalies of the kidney and urinary tract
ESRD	end-stage renal disease
SWL	shockwave lithotripsy
PNL	percutaneous nephrolithotomy
RIRS	retrograde intrarenal surgery
EAU	European Association of Urology
HU	Hounsfield unit
KUB	kidney, ureter, and bladder
USG	ultrasonography
NCCT	non-contrast-enhanced computed tomography
SF	stone-free
D/J	Double-J
FANS	flexible and navigable suction ureteral access sheath
TFL	thulium fiber laser
RUSS	Resorlu-Unsal Stone Score
IPA	infundibulopelvic angle
